# Autochthonous Melioidosis in Humans, Madagascar, 2012 and 2013

**DOI:** 10.3201/eid2010.131524

**Published:** 2014-10

**Authors:** Benoit Garin, Innocente Djaomazala, Natasha Dubois-Cauwelaert, Mahafaly, Vaomalala Raharimanga, Fidiarivony Ralison, Perlinot Herindrainy, Nivosoa C. Andriamalala, Derek S. Sarovich, Mark Mayo, Mirjam Kaestli, Bart J. Currie

**Affiliations:** Pasteur Institute, Antananarivo, Madagascar (B. Garin, N. Dubois-Cauwelaert, V. Raharimanga, P. Herindrainy);; Androva University Hospital, Mahajanga, Madagascar (I. Djaomazala, Mahafaly, F. Ralison, N.C. Andriamalala);; Menzies School of Health Research, Darwin, Northern Territory, Australia (D.S. Sarovich, M. Mayo, M. Kaestli, B.J. Currie);; Royal Darwin Hospital, Darwin (D.S. Sarovich, M. Mayo, M. Kaestli, B.J. Currie)

**Keywords:** Melioidosis, human case, Burkholderia pseudomallei, Madagascar, Africa, Indian Ocean, MLST, bacteria

## Abstract

Melioidosis is an often fatal infectious disease affecting humans and animals in the tropics. Only sporadic cases have been reported from Africa and the Indian Ocean region. We describe 2 confirmed autochthonous cases of human melioidosis in Madagascar, both from novel genotypes of *Burkholderia pseudomallei*.

Melioidosis is an often fatal infectious disease caused by the soil bacterium *Burkholderia pseudomallei*. Incidence rates are increasing in regions of Southeast Asia and northern Australia to which it is endemic, and cases are increasing from the tropics worldwide (*1*). Little is known about the epidemiology of melioidosis in the Indian Ocean region and Africa, where only sporadic cases are reported (*2*–*5*). We report 2 melioidosis cases in residents of the city of Mahajanga, the capital of the Mahajanga Province of Madagascar (Figure). These cases were identified within an ongoing project to document melioidosis in Madagascar conducted by the Pasteur Institute in Antananarivo.

**Figure:**
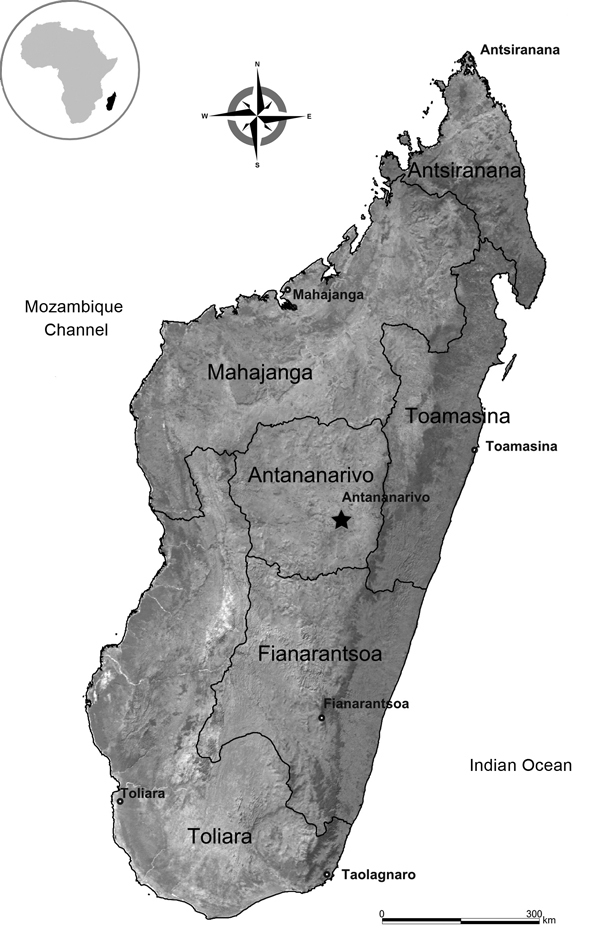
Map of Madagascar. Inset: Africa showing location of Madagascar.

Mahajanga, a favorite tourist destination in Madagascar, is located on the country’s northwestern coast on the Mozambique Channel (15°43′S, 46°18′E), 550 km from Antananarivo, the capital of Madagascar. Its population is 200,000 persons.

## Case Reports

Case-patient 1 was a 52-year-old male rural rice farmer, who had diabetes. He was admitted to Androva University Hospital in Mahajanga in July 2012 with systemic sepsis, from which he died 3 days later despite treatment with ceftriaxone. Chest radiographs showed opacity in the lower left lung and pleural effusion. Abdominal ultrasound showed hepatomegaly and splenomegaly with small hypoechogenic lesions in the spleen consistent with abscesses. Culture media used to recover organisms from the blood were Ashdownagar supplemented with gentamicin 4 mg/L (*6*), chocolate agar, and blood agar incubated for 24 h at 37°C after inoculation. A blood culture (Hemoline Performance Diphasique, bioMérieux, Marcy l’Etoile, France) taken the day after admission was positive for *B. pseudomallei*; *B. pseudomallei* was suspected 8 days later on the basis of a biochemical phenotype (82% identity) using API20NE, 1154576 (bioMérieux).

Case-patient 2 was a 45-year-old male rice, sugar cane, and tobacco farmer, who had diabetes. He was admitted to Androva University Hospital in May 2013 with a recurrent fever and a history of furunculosis for several months. A week after admission, his condition deteriorated; he had progressive sepsis and hepatic failure despite therapy with ceftriaxone, ciprofloxacin, metronidazole, and gentamicin. Ultrasound of the abdomen showed hepatomegaly with a multinodular appearance and splenomegaly. Four blood samples were cultured on different days during his illness. Gram-negative colonies showed an API20NE biochemical profile of 1156576, suggesting *B. pseudomallei* (99% identity). After melioidosis was presumptively diagnosed, the patient was immediately treated with ceftazidime but he died 24 hours later, 2 weeks after admission.

DNA extracted from the presumptive *B. pseudomallei* isolates was positive by real-time PCR (*7*). Multilocus sequence typing (MLST) was performed as previously described (*8*). MLST demonstrated 2 novel allele combinations of 4, 12, 3, 2, 5, 2, 1 for case-patient 1 and 4, 12, 34, 1, 5, 2, 1 for case-patient 2. Sequences were submitted to the MLST database (http://bpseudomallei.mlst.net/) and have been assigned the sequence types (ST) 1053 and 1054, respectively.

## Conclusions

The fatal outcome of these 2 case-patients reflects the reality of medical care prevailing in Madagascar and much of Africa, where hospital resources are limited and the capability for diagnosing melioidosis as part of routine laboratory practice does not exist. During 1936–2012, *B. pseudomallei* was isolated on rare occasions from Madagascar. In 1936, the Whitmore bacillus was isolated from a submaxillary node of a slaughtered pig by G. Girard, who was working at the Pasteur Institute in Madagascar. This was the first time this bacterium was identified in Africa (*9*). In 1982, M. Galimand and A. Dodin, working in the Whitmore bacillus laboratory at the Pasteur Institute in Paris, published a review of the distribution of *Pseudomonas pseudomallei* worldwide (*10*) and reported that in 1977 this bacterium was found in the soil of a zoo and pig farm in Antananarivo. In 2004, a French patient coming from Madagascar was diagnosed in La Réunion with melioidosis (*11*). He lived in Mahajanga city, but 25 years earlier had lived in Vietnam, where melioidosis is endemic. In 2005, melioidosis was diagnosed in a second French patient in La Réunion that was also attributed to infection in Madagascar; this person lived in Antananarivo, but disease onset occurred during a stay in Mahajanga (*12*). In 2013, melioidosis was diagnosed in Brussels in a third French patient living in Belgium who had returned from Mahajanga (*13*). His history did not reveal travel to any other melioidosis-endemic areas, and his infection was likely to have been acquired in Mahajanga.

Mahajanga has a tropical savanna climate; the rainy season is mostly from December to April. The 2 cases reported here occurred at the end of the rainy season; onset of all 3 published cases in French visitors occurred during March (2004, 2005, 2013). Before the 2 cases reported here from Madagascar, the only confirmed autochthonous case of melioidosis from Africa of which we were aware was in a 16-month-old boy from a remote village in rural Malawi who sought care in March 2011 (*3*). That case was also able to be diagnosed because of enhanced laboratory facilities associated with research support. In Mauritius, the first autochthonous confirmed case of melioidosis was diagnosed in 2004 (*4*).

Genotyping of the 2 *B. pseudomallei* strains reported here showed novel STs for both. Furthermore, the MLST result from the third French case-patient recently reported (*13*) demonstrated another novel allelic profile, 4, 1, 3, 2, 5, 2, 1, which has been designated ST1043. ST1043 is a single locus variant of ST1053 from case-patient 1 and ST1054 from case-patient 2 is a single locus variant of ST319, which is represented only by a single *B. pseudomallei* isolate cultured from the 2004 Mauritius case-patient (*4*). The B. pseudomallei isolate cultured from the recently reported Spanish traveler to Africa was ST879 (*2*), which is very divergent from STs 1043, 1053, and 1054, and suggests that her infection might have been acquired in 1 of the 14 West African countries she traveled through, rather than in Madagascar, which she also visited.

Although these genotype data are consistent with the findings recently reported from Africa that suggested a possible recent ancestor for strains from Malawi and Kenya, an alternative hypothesis is that the diversity and novel STs seen in Madagascar might represent more ancient origins for *B. pseudomallei* in Africa. Phylogeographic reconstruction of *B. pseudomallei* genomes has supported an Australian origin for *B. pseudomallei*, with a possible single introduction event into Southeast Asia, possibly during the last ice age when low sea levels created land bridges between what are now islands in the Malay Archipelago (*14*). Nevertheless, studies by Pearson et al. focused on strains from Australia and Southeast Asia and included few strains from the rest of the world. Without detracting from that hypothesis of spread from Australia to Southeast Asia, we suggest that a prior hypothesis of more ancient origins of *B. pseudomallei* (*15*) also could explain the novel and diverse genotypes of *B. pseudomallei* in Africa and in the Americas (*1*). Ongoing whole-genome sequencing of multiple isolates from diverse locations should resolve the origins and global dispersal patterns of *B. pseudomallei*.

In summary, 2 fatal cases of melioidosis in farmers who had diabetes confirm that melioidosis is endemic in Madagascar. Difficulties with diagnosis and treatment of melioidosis in less developed countries, such as Madagascar, highlight the need for support for improved laboratory services and for further collaborative studies to elucidate the epidemiology of melioidosis in regions such as Africa where its presence is suspected but where data are very limited.

## References

[1] Wiersinga WJ, Currie BJ, Peacock SJ. Melioidosis. N Engl J Med. 2012;367:1035–44. 10.1056/NEJMra1204699 22970946

[2] Morosini MI, Quereda C, Gil H, Anda P, Núñez-murga M, Cantón R. Melioidosis in traveler from Africa to Spain. Emerg Infect Dis. 2013;19:1656–9. 10.3201/eid1910.121785 24047798 PMC3810733

[3] Katangwe T, Purcell J, Bar-Zeev N, Denis B, Montgomery J, Alaerts M, Human melioidosis, Malawi, 2011. Emerg Infect Dis. 2013;19:981–4. 10.3201/eid1906.120717 23735189 PMC3713813

[4] Issack MI, Bundhun CD, Gokhool H, Case T. Melioidosis in Mauritius. Emerg Infect Dis. 2005;11:139–40. 10.3201/eid1101.040605 15705340 PMC3294329

[5] Currie BJ. Dance D a B, Cheng AC. The global distribution of *Burkholderia pseudomallei* and melioidosis: an update. Trans R Soc Trop Med Hyg. 2008;102(Suppl 1):S1–4. 10.1016/S0035-9203(08)70002-6 19121666

[6] Peacock SJ, Chieng G, Cheng AC, Dance DAB, Amornchai P, Wongsuvan G, Comparison of Ashdown’s medium, Burkholderia cepacia medium, and Burkholderia pseudomallei selective agar for clinical isolation of *Burkholderia pseudomallei.* J Clin Microbiol. 2005;43:5359–61. 10.1128/JCM.43.10.5359-5361.2005 16208018 PMC1248505

[7] Novak RT, Glass MB, Gee JE, Gal D, Mayo MJ, Currie BJ, Development and evaluation of a real-time PCR assay targeting the type III secretion system of *Burkholderia pseudomallei.* J Clin Microbiol. 2006;44:85–90 . 10.1128/JCM.44.1.85-90.2006 16390953 PMC1351940

[8] Godoy D, Randle G, Simpson AJ, Aanensen DM, Pitt TL, Kinoshita R, Multilocus sequence typing and evolutionary relationships among the causative agents of melioidosis and glanders, *Burkholderia pseudomallei* and *Burkholderia mallei.* J Clin Microbiol. 2003;41:2068–79. 10.1128/JCM.41.5.2068-2079.2003 12734250 PMC154742

[9] Girard G. Can pigs be a healthy carrier of Whitmore’s bacillus? [in French]. Bull Soc Pathol Exot. 1936;29:712–6.

[10] Galimand M, Dodin A. A review of melioidosis worldwide [in French]. Bull Soc Pathol Exot. 1982;75:375–83. 7172358

[11] Martinet O, Soo AMP, Knezynski M, Schlossmacher P, Jaffar-Bandjee C, Gaüzère BA. Melioidosis: regarding a case acquired in Madagascar and two nosocomial cases [in French]. Bull Soc Pathol Exot. 2004;97:366–70.

[12] Borgherini G, Poubeau P, Paganin F, Picot S, Michault A, Thibault F, Melioidosis: an imported case from Madagascar. J Travel Med. 2006;13:318–20. 10.1111/j.1708-8305.2006.00050.x 16987131

[13] Rossi C. Melioidosis—Belgium ex Madagascar. ProMed. 2013 May 3. http://www.promedmail.org, archive no. 20130503.1687746.

[14] Pearson T, Giffard P, Beckstrom-sternberg S, Auerbach R, Hornstra H, Tuanyok A, Phylogeographic reconstruction of a bacterial species with high levels of lateral gene transfer. BMC Biol. 2009;7:78. 10.1186/1741-7007-7-78 19922616 PMC2784454

[15] Cheng AC, Godoy D, Mayo M, Gal D, Spratt BG, Currie BJ. Isolates of *Burkholderia pseudomallei* from northern Australia are distinct by multilocus sequence typing, but strain types do not correlate with clinical presentation. J Clin Microbiol. 2004;42:5477–83. 10.1128/JCM.42.12.5477-5483.2004 15583269 PMC535284

